# Gastric Outlet Obstruction as a First Symptom of a Non‐Muscle Invasive Bladder Cancer (NMIBC) Progression—A Case Report

**DOI:** 10.1002/cnr2.70077

**Published:** 2024-12-12

**Authors:** Duje Apostolski, Florian Roitner

**Affiliations:** ^1^ Department of Internal Medicine Hospital St. Josef Braunau Braunau am Inn Austria

**Keywords:** gastric outlet obstruction, metastatic bladder cancer, NMIBC, non‐muscle invasive bladder cancer, peritoneal carcinomatosis

## Abstract

**Background:**

Metastatic disease of a urinary bladder cancer localized in the upper abdomen is very rare. This case report describes the first patient with a urinary bladder cancer progression, initially presenting as a gastric outlet obstruction due to peritoneal carcinomatosis.

**Case:**

We present the case of a 78‐years‐old male patient who was admitted to Hospital St. Josef Braunau in Austria with persistent vomiting. In the medical history, the most prominent finding was a diagnosed high‐risk NMIBC. At the time, patient was between 2. and 3. BCG maintenance instillation cycle, following two transurethral resections. Routine follow‐up cystoscopy 1 month before admission to our department showed no evidence of disease recurrence. Due to the therapy resistant vomiting, gastroscopy was performed, revealing duodenal stenosis without mucosal changes. Subsequently performed abdominal CT‐scan showed homogenous swelling of the mesenteric fat tissue around duodenum, spreading retroperitoneal to both kidneys. In the absence of the typical peritoneal carcinomatosis features, the finding was firstly described as an inflammation of mesenteric fat or *panniculitis mesenterialis*. Further deterioration of patient's condition and later occurred bilateral hydronephrosis raised a suspicion of peritoneal carcinomatosis. Consequently, conducted laparoscopic exploration confirmed the suspicion describing the tissue conglomerate typical for peritoneal carcinomatosis surrounding the duodenum. Pathohistological analysis of taken samples proved urothelial cancer cells, confirming the diagnosis of metastatic bladder cancer disease.

**Conclusion:**

This case report presents a very unusual presentation of metastatic urinary bladder cancer that could help clinicians to consider this diagnosis when encountering similar clinical features.

**List of abbreviations:**
BCG: bacillus Calmette‐Guerin
CIS: “carcinoma in situ”
CT: computer tomography
EAU: European Association of Urology
GI: gastrointestinal
MIBC: muscle invasive bladder cancer
NMIBC: non muscle invasive bladder cancer

## 1 Background

Bladder cancer is the 10th most common cancer in the world and its incidence is steadily rising worldwide, especially in developing nations. The worldwide standardized incidence and mortality rates in males are 9.5 and 3.3 per 100 000 respectively, being about four times higher than those rates among females [1].

The main risk factor appears to be smoking, followed by etiologic factors of cardiovascular diseases like obesity, hyperglycaemia, hypertension, and hypertriglyceridemia [2].

Transitional cell or urothelial cell carcinoma accounts for more than 90% of all bladder cancers out of which 70% are non‐muscle invasive (NMIBC) at the time of diagnosis [3]. Different factors such as age of the patient, tumor size, presence of CIS and histological grade amongst others influence the ability of the tumor to progress in muscle invasive bladder cancer (MIBC). The newest guidelines suggest dividing the risk into four groups‐ low, intermediate, high, and very high. High‐ and very high‐risk NMIBC have a recurrence rate of 70% with 15%–40% risk of progression as compared to less than 5% in 5 years for low‐risk type [4].

The first choice of treatment for high risk NMIBC after the transurethral resection is intravesical bacillus Calmette‐Guerin (BCG) instillation therapy. In selected cases of BCG non‐responders, a radical cystectomy should be considered [4].

Most common metastatic sites of bladder are bones, pelvis, and lungs [5]. Involvement of peritoneum is considered frequent, especially in more developed stages of the disease [6]. However, mechanism of peritoneal carcinomatosis development in urinary bladder cancer is still not fully understood [7].

Diagnosis of peritoneal carcinomatosis generally presents a challenge to clinicians to this day. Most common signs of peritoneal carcinomatosis from different malignant diseases are abdominal free fluid and bowel obstruction. In many cases it can be asymptomatic, appearing as an accidental finding [8].

In this paper, we present the first case of gastric outlet obstruction due to peritoneal carcinomatosis being the leading symptom of a non‐muscle invasive bladder cancer (NMIBC) progression.

### 1.1 Case Presentation

A 78‐year‐old male patient was presented in an emergency department of Hospital St. Josef Braunau in Austria in September 2021. With extensive vomiting without abdominal pain. Reportedly, symptoms had been lasting few days without signs of improvement.

History of smoking, high blood pressure, atrial fibrillation, and hyperlipidemia were known from the patient's history. However, the most prominent finding was a performed transurethral bladder resection 13 months ago in a nearby hospital. Official histology finding after the resection described a high grade pT1 and CIS of a urinary bladder carcinoma. Because of the absence of muscle invasion and presence of carcinoma in situ (CIS), the tumor was graded as high‐risk non‐muscle invasive bladder cancer (high‐risk NMIBC). Being graded as high‐risk, an additional resection to exclude a residual disease was performed 1 month later, showing a residual pTa urinary carcinoma. As the next step, therapy with BCG was introduced. Since the first control cystoscopy after the BCG induction (about 8 months before admission at our hospital) there were no histological signs of disease. Last routine cystoscopy control took place 2 months before the first admission at our hospital and again showed only normal findings. At that time, patient was between 2. and 3. BCG maintenance instillation cycle.

Standard blood and urine laboratory tests, as well as standing abdominal x‐ray were performed. All the laboratory findings were in normal range and abdominal x‐ray did not show any bowel obstruction or hollow organ perforation. Patient later also mentioned that he occasionally suffers from obstipation. As the vomiting improved under antiemetic therapy and colonoscopy showed only few benign polyps, patient was at first discharged from hospital after 3 days with obstipation as the main diagnosis.

**FIGURE 1:**
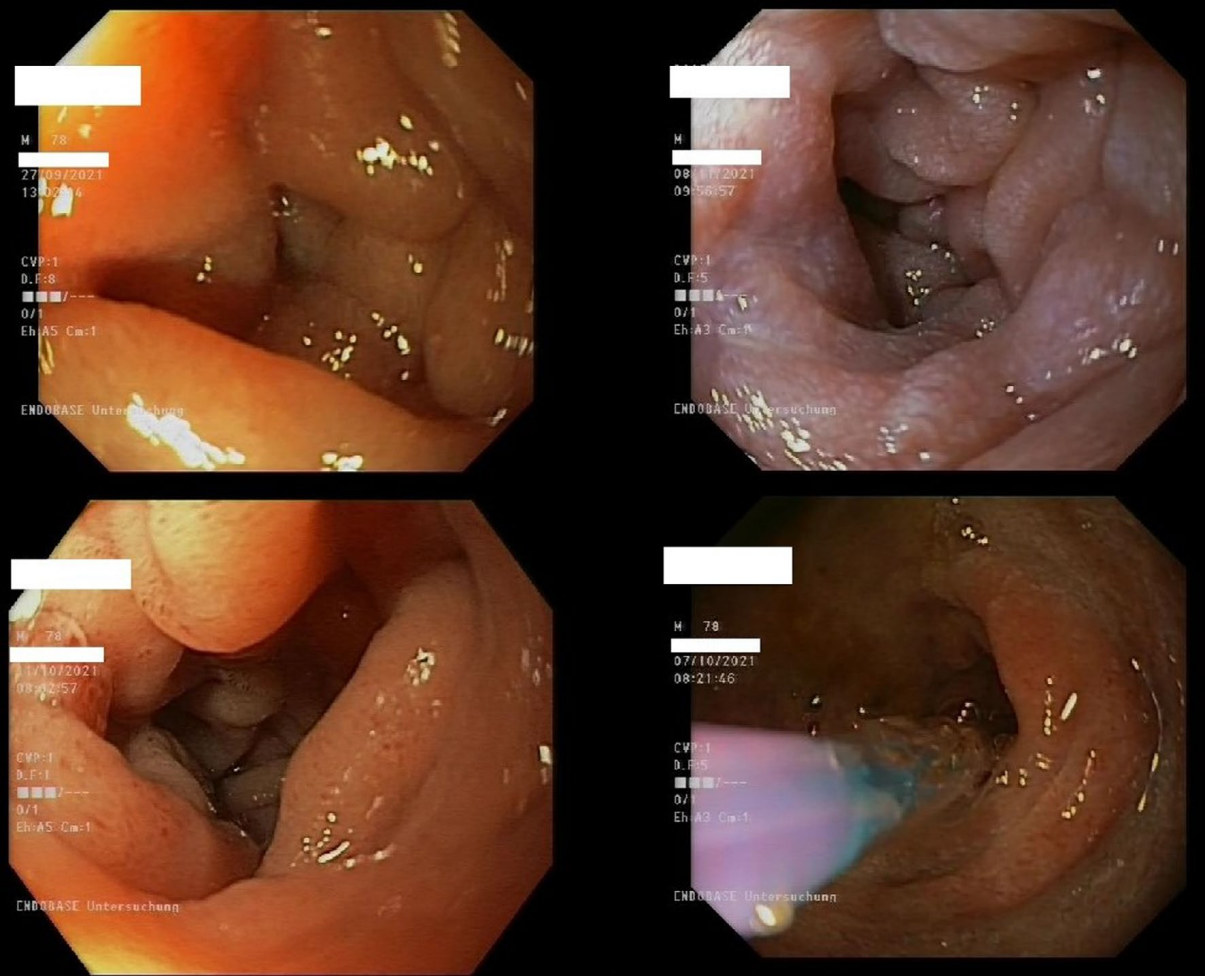
Duodenal stenosis in different stages of the disease during upper GI endoscopy. The lower right figure shows the part of the balloon dilatation process.

After 2 weeks, patient was again admitted with even more frequent vomiting, again without abdominal pain or signs of acute abdomen. All laboratory and radiographic findings were again without pathological features. This time vomiting was therapy resistant, so we decided to perform a gastroscopy. It showed distended stomach filled with fluid despite approximately 12 h of fasting time, indicating possible obstruction. The cause was found already in proximal part of duodenum, where a total lumen stenosis could be described. Duodenal wall was closing equally from all sides, without visible mucosal damage, suggesting extraluminal cause of the stenosis (Figure 1). Biopsy samples were taken and immediately abdominal CT scan for further diagnostic work‐up was performed.

**FIGURE 2:**
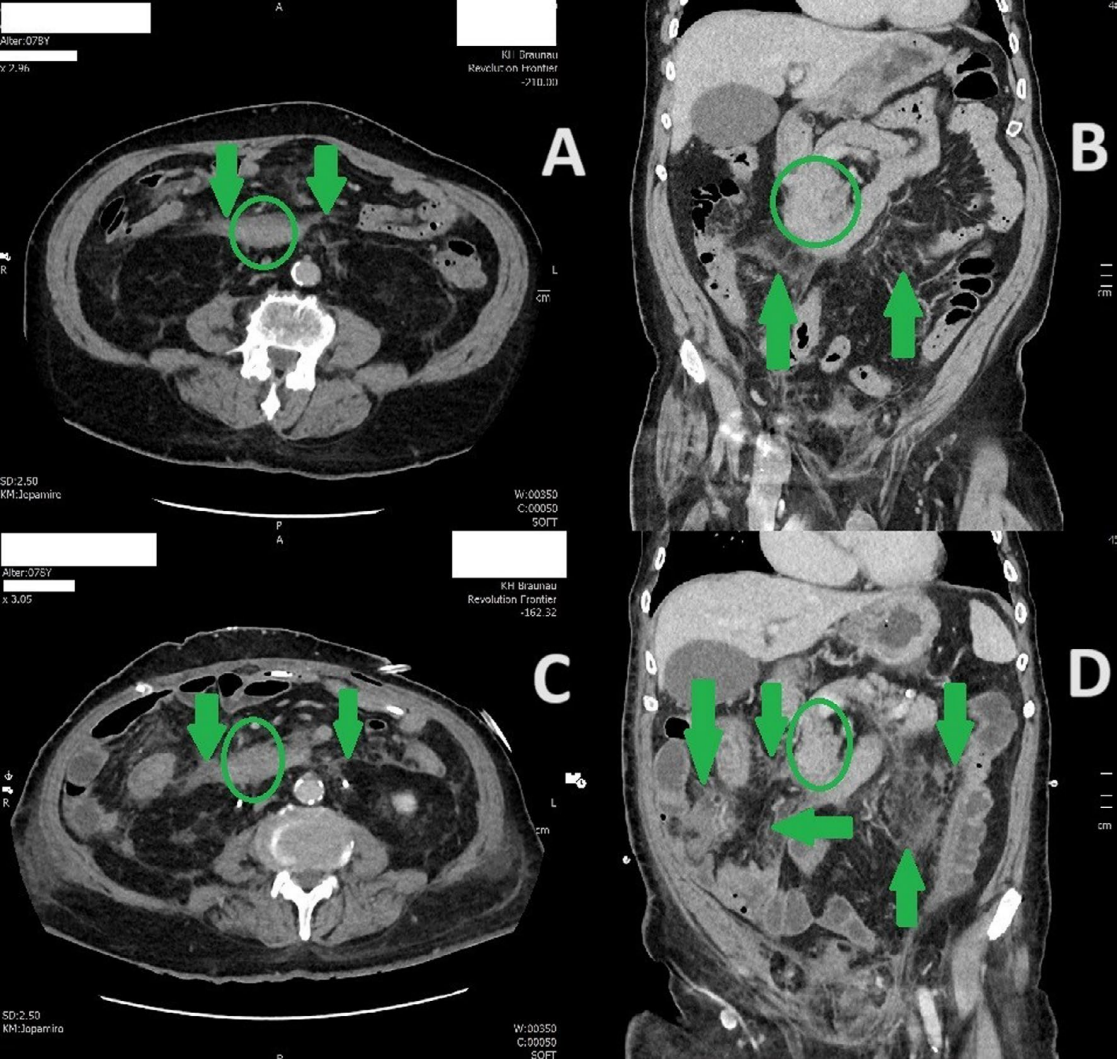
(A, B) First CT abdomen with contrast in axial and coronal plane showing hyperdense structure surrounding duodenum (encircled area) with lateral retroperitoneal spreading (green arrows). (C, D) Last CT scan before chemotherapy showing progression of the finding‐ in the meantime the lesion was proved to be peritoneal carcinomatosis.

In the first CT scan radiologist described homogenous swelling of a fat tissue surrounding duodenum, spreading retroperitoneal to both kidneys, making the differential diagnosis of inflamed mesenterial fat tissue or *panniculitis mesenterialis* (Figure 2A,B). In the meantime, biopsy samples of duodenal mucosa showed no specific signs of pathology. Therapeutically, endoscopic balloon‐dilatation of duodenal stenosis was performed. It helped to relieve the vomiting and ensure regular medication intake. Patient was discharged after 3 weeks with the diagnosis of a gastric outlet obstruction due to *panniculitis mesenterialis* of unclear origin and was scheduled for control gastroscopy in 3 weeks. The control gastroscopy showed a passable duodenum, still with distinct swelling of the mucosa.

**FIGURE 3:**
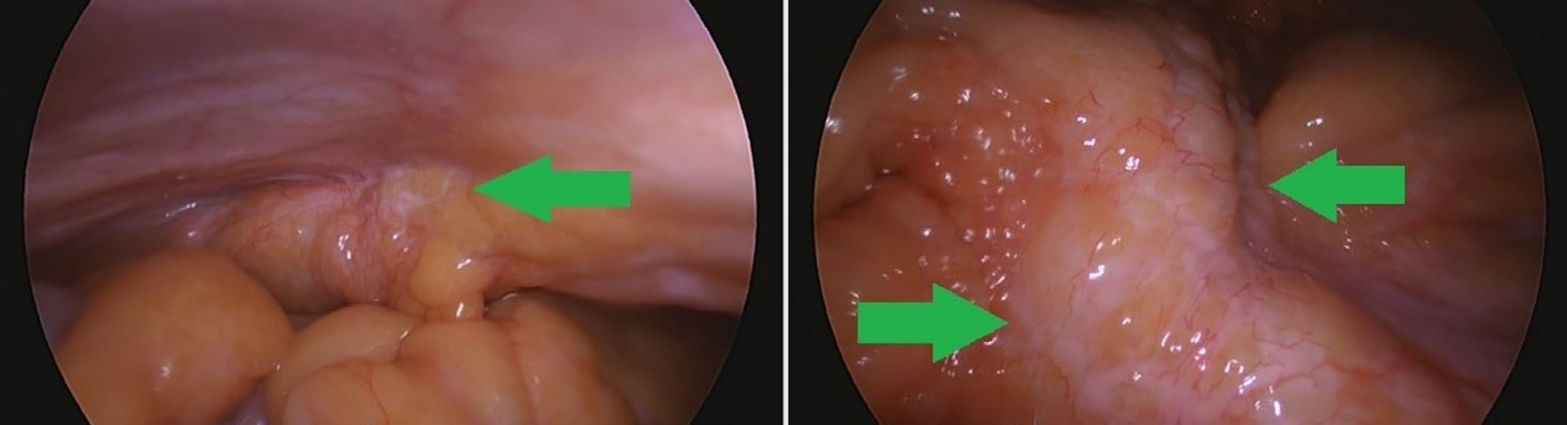
Adhesions due to peritoneal carcinomatosis (left, green arrow) and tissue conglomerate on the fat tissue surrounding duodenum (right, green arrows) documented during explorative laparoscopy.

Nevertheless, in the following weeks the patient's state continued to deteriorate, manifested by further weight loss and general weakness. Laboratory findings this time showed worsening of kidney function and the orientating abdominal ultrasound showed newly occurred hydronephrosis. Patient was again admitted and control native abdominal CT scan was obtained, confirming suspected hydronephrosis. Without contrast, progression of mesenterial fat tissue inflammation could not be assessed. Eventually, considering rapid deterioration of the patient's state and probable progression of the previous findings in the upper abdomen due to newly occurred hydronephrosis, peritoneal carcinomatosis was suspected. Consequently, laparoscopic exploration was conducted at the end of the November 2021 as a last diagnostic modality in order to clarify the above mentioned clinical and radiological findings. The operation report described conglomerate lesions typical for peritoneal carcinomatosis surrounding the fat tissue around duodenum and spreading across upper abdomen (Figure 3). Following pathohistological analysis of the described lesion showed urothelial cancer cells. During the procedure, gastrojejunostomy was also performed to prevent further vomiting.

Diagnosis of the progression of urothelial cell bladder cancer was confirmed. Last abdominal CT scan with contrast as a part of a staging assessment before chemotherapy provided imaging evidence peritoneal carcinomatosis progression (Figure 2C,D). Palliative chemotherapy with cisplatin and gemcitabine was introduced in the second half of the December 2021. Unfortunately, further progression of the disease resulted in introduction of the best supportive care and end of life treatment in February 2022.

## 2 Discussion

Peritoneal carcinomatosis is the most common malignant process of the peritoneal cavity, usually being part of the progressive intraabdominal tumor disease (gastric, colorectal and gynaecological malignancies). Its diagnosis presents a real challenge in an everyday practice [9].

According to literature, the most typical imaging signs of peritoneal carcinomatosis are multiple, mostly nodular, irregular and inhomogeneous lesions at the sites of natural peritoneal fluid stasis: peritoneal reflexion of the pelvis, lower small bowel mesentery, sigmoid mesocolon, right paracolic gutter, right subphrenic space and the omentum. Furthermore, other common findings are also ascites and infiltration of fat tissue [9]. In present case, changes in mesenterial fat tissue were also described. However, these changes were rather homogenous, therefore repeatedly described as a possible inflammation of the mesenteric fat tissue (*panniculitis mesenterialis*).

Moreover, the site of the lesion was exclusively in upper abdomen, which is very atypical for a metastatic site of the urinary bladder cancer. We found only two papers describing urinary bladder cancer metastatic disease involving upper abdomen. Both papers described direct involvement of a duodenum presenting with expected complications: upper GI bleeding and small bowel obstruction, respectively [10, 11]. Metastatic potential of bladder cancer is still unknown, especially regarding peritoneal carcinomatosis. There are some studies that have proven the immense ability of bladder cancer to invade peritoneum, due to its special anatomical position [7]. However, this applies only to a local disease progression in the small pelvis, whereas ability to invade other parts of peritoneal cavity remains uncertain.

Most common symptoms of peritoneal carcinomatosis are small bowel obstruction and ascites [9]. Our patient indeed presented with a subtype of the bowel obstruction. Nevertheless, site of the obstruction was extremely surprising, as we have not found any data describing the gastric outlet obstruction as a leading symptom of peritoneal carcinomatosis. Causes of gastric outlet obstruction can originate from pathologic changes that occur inside or outside of the stomach and duodenum lumen. Outside causes are mostly malignant tumors of adjacent organs pressing the lumen [12]. However, in our case the pressure on duodenum was caused by inflammation and swelling of the surrounding mesenteric fat tissue due to metastatic changes.

The clinical picture and findings were very unusual and it was hard to make a diagnosis of the metastatic bladder cancer despite the fact that the patient had many risk factors for recurrence and would be classified as a very‐high risk patient according to the newest EUA guidelines for NMIBC. On top of all above mentioned, knowing that the cystoscopy 1 month before admission showed no signs of urinary bladder cancer, finding of an advanced metastatic disease was utterly surprising and unprecedented.

## 3 Conclusion

This case report describes a very unusual presentation of a well‐known disease. It might help the clinicians to consider the diagnosis of peritoneal carcinomatosis and metastatic urinary bladder cancer disease when coming across similar clinical presentation and medical history.

### 3.1 Timeline

(Figure 4).

**FIGURE 4:**
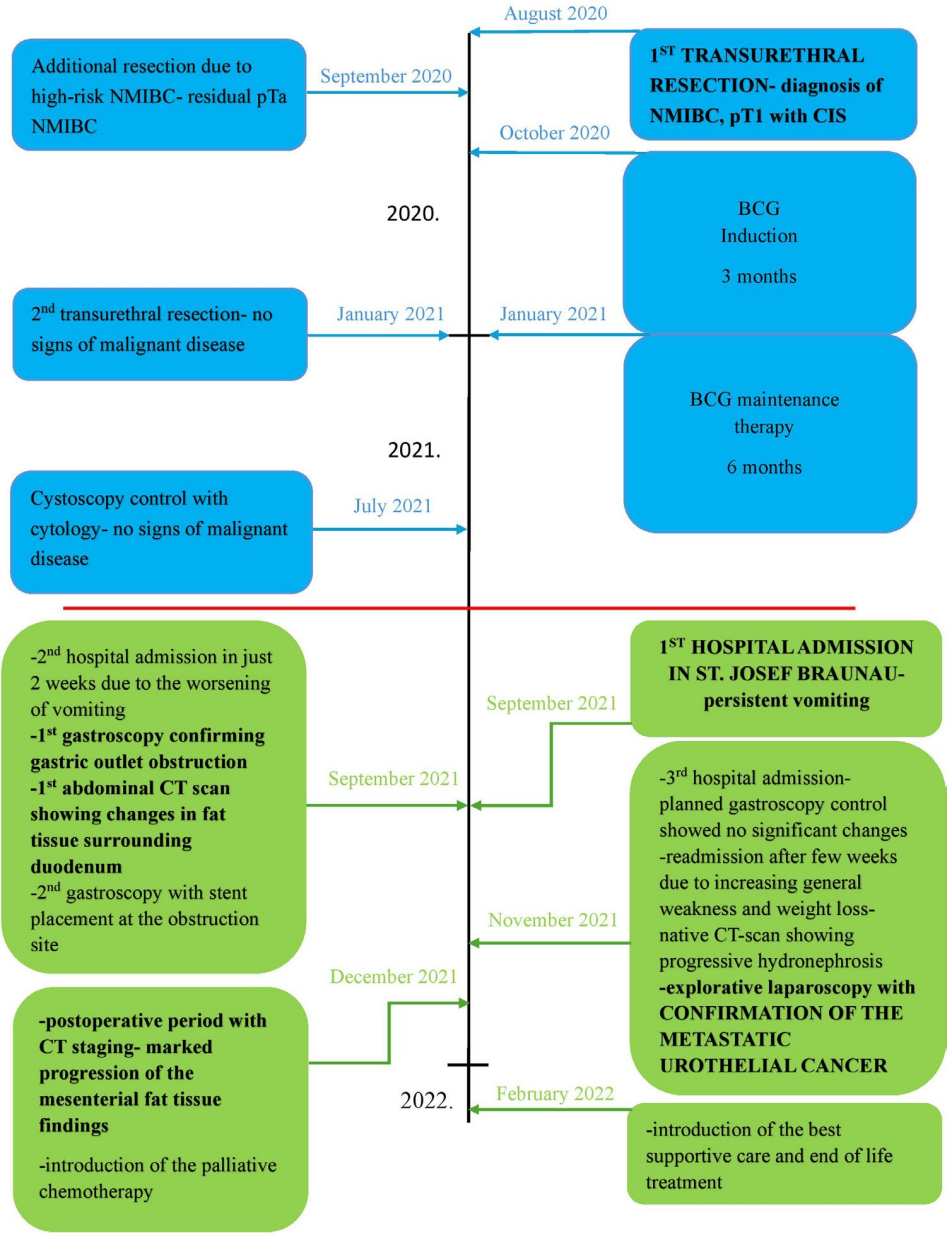
Case report timeline showing detailed sequence of the events from the first diagnosis of urothelial carcinoma. Red line is dividing the treatments that had been done in the nearby hospital, additionally enhanced with the blue color. Treatment at our hospital is marked with green.

## Author Contributions

**Duje Apostolski:** conceptualization, investigation, methodology, validation, formal analysis, data curation, resources, writing – original draft. **Florian Roitner:** conceptualization, methodology, validation, writing – review and editing.

## Consent

Consent was obtained from the family (patient's wife) as the patient unfortunately passed away during writing of the case report.

## Conflicts of Interest

The authors declare no conflicts of interest.

## Data Availability

The data that support the findings of this study are available from the corresponding author upon reasonable request.
